# The detection of microRNA associated with Alzheimer's disease in biological fluids using next-generation sequencing technologies

**DOI:** 10.3389/fgene.2013.00150

**Published:** 2013-08-08

**Authors:** Lesley Cheng, Camelia Y. J. Quek, Xin Sun, Shayne A. Bellingham, Andrew F. Hill

**Affiliations:** ^1^Department of Biochemistry and Molecular Biology, The University of MelbourneMelbourne, VIC, Australia; ^2^Department of Biochemistry and Molecular Biology, Bio21 Molecular Science and Biotechnology Institute, The University of MelbourneMelbourne, VIC, Australia; ^3^Melbourne Brain Centre, Mental Health Research Institute, The University of MelbourneMelbourne, VIC, Australia

**Keywords:** microRNA, biological fluids, exosomes, Alzheimer's disease, deep sequencing

## Abstract

Diagnostic tools for neurodegenerative diseases such as Alzheimer's disease (AD) currently involve subjective neuropsychological testing and specialized brain imaging techniques. While definitive diagnosis requires a pathological brain evaluation at autopsy, neurodegenerative changes are believed to begin years before the clinical presentation of cognitive decline. Therefore, there is an essential need for reliable biomarkers to aid in the early detection of disease in order to implement preventative strategies. microRNAs (miRNA) are small non-coding RNA species that are involved in post-transcriptional gene regulation. Expression levels of miRNAs have potential as diagnostic biomarkers as they are known to circulate and tissue specific profiles can be identified in a number of bodily fluids such as plasma, CSF and urine. Recent developments in deep sequencing technology present a viable approach to develop biomarker discovery pipelines in order to profile miRNA signatures in bodily fluids specific to neurodegenerative diseases. Here we review the potential use of miRNA deep sequencing in biomarker identification from biological fluids and its translation into clinical practice.

## Introduction

The pathophysiological process of neurodegenerative disorders such as Alzheimer's disease (AD) begins well before the diagnosis of clinical dementia. AD is characterized pathologically by the presence of insoluble plaques and tangles composed of beta-amyloid (Aβ) formed by sequential amyloid precursor protein (APP) proteolysis and hyperphosphorlyated Tau (pTau) proteins (Hardy and Allsop, 1991; Walsh and Selkoe, 2004; Cole and Vassar, 2008). The accumulation of Aβ has been the major focus of AD research and has been shown to interfere with long term potentiation which is required for neuronal signaling, and is implicated in pro-apoptotic signaling leading to neuronal loss (Chapman et al., 1999; Roth, 2001). The majority of AD patients are asymptomatic during the pre-clinical stages of the pathological process which is believed to be a period of approximately 17 years (Villemagne et al., 2013). Therefore, early diagnosis of AD is required before or during the pre-clinical phase in order that therapeutic intervention, or the use of disease modifying drugs, can be administered.

The main biomarker targets currently employed for AD diagnosis are measurements of Aβ and pTau (Hansson et al., 2010; Prvulovic and Hampel, 2011; Watt et al., 2011; Rosenmann, 2012). MicroRNA (miRNA) are a class of non-coding RNA of approximately 22 nucleotides in length, and are known to regulate post-translational transcription. Expression profiling of miRNA levels represents a new class of potential biomarkers that are currently being investigated for the diagnosis of a number of diseases (Skog et al., 2008; Taylor and Gercel-Taylor, 2008; Baraniskin et al., 2012b; Geekiyanage et al., 2012; Jones et al., 2012). miRNAs are derived from RNA hairpins comprising of precursor miRNA and processed by endoribonucleases (Dicer and Drosha) to form mature miRNA fragment (Krol et al., 2010). The mature miRNA is incorporated into the RNA-induced silencing complex (RISC) which binds to complementary sites in the 3′ untranslated region of their mRNA targets resulting in downregulation of gene expression (He and Hannon, 2004). They can be secreted into biological fluids where they can be detected and profiled using methods including quantitative real-time PCR (qRT-PCR), microarrays, and more recently by deep sequencing technologies.

Here, we review the current literature to highlight the diagnostic potential to screen for neurodegeneration using gene expression profiling in biological fluids. In particular, we have focused on the potential of profiling miRNA expression associated with AD and evaluate the current deep sequencing platforms suitable for biomarker discovery including the implementation into clinical diagnostic laboratories.

## Brain-associated micrornas and their detection in biological fluids

It has been revealed that the highest expression of tissue specific miRNA is found in the brain (Babak et al., 2004; Sempere et al., 2004; Schonrock et al., 2010). The significance of miRNA and their conclusive biological functions were gradually discovered using knockout mouse models (Schaefer et al., 2007). For example, Dicer knockout mice have been shown to deregulate miRNA processing, leading to defects in neuronal development and underdevelopment of the brain, demonstrating a role for miRNA in neurogenesis (Schaefer et al., 2007; Kawase-Koga et al., 2009; Huang et al., 2010). The first reports translating early miRNA studies to the brain observed a number of brain enriched miRNA such as miR-9, miR-29a, miR-125, miR-128, miR-134, and miR-137 (Table 1). With respect to AD, a number of deregulated miRNA have been identified to correlate with disease, including miR-9, miR-20a, and miR-132 (Makeyev et al., 2007; Cogswell et al., 2008; Hebert et al., 2009, 2012). While, synthetic miRNA precursors, miR-20a, miR-17-5p, and miR-106b, when co-transfected in HeLa cell lines, inhibited APP protein translation (Hebert et al., 2009). Highly abundant and brain enriched miRNAs found to be deregulated in AD models (human and mice models) are summarized in Table 1.

**Table 1:** **Highly abundant miRNAs that are deregulated in AD and detected in biological fluids**.

**miRNA**	**Homeostatic functions**	**References**	**Species**	**Deregulated in AD**	**References**	**Species**	**Detected in biological fluids**	**References**	**Species**
miR-9	Neuronal differentiation	Saunders et al., 2010; Shibata et al., 2011; Tan et al., 2012	Mouse	Down regulated in the presence of Aβ	Cogswell et al., 2008; Schonrock et al., 2010, 2012	Human	Plasma, urine, CSF	Melkonyan et al., 2008; Alexandrov et al., 2012; Geekiyanage et al., 2012; Patz et al., 2013	Human
miR-29 (a/b)	Neuronal maturation and apoptosis	Kole et al., 2011	Mouse	Down regulation affects BACE1 and Aβ levels	Hebert et al., 2008	Human, mouse	Plasma, urine, CSF	Geekiyanage et al., 2012; Wang et al., 2012b; Patz et al., 2013	Human
miR-124	Neuronal differentiation	Smirnova et al., 2005; Caltagarone et al., 2007; Hebert et al., 2008	Mouse, chick embryo, human	Targets BACE1	Fang et al., 2012	Rat (PC12)	Urine, CSF	Melkonyan et al., 2008; Patz et al., 2013	Human
miR-128	Neuronal differentiation and development	Smirnova et al., 2005; Schonrock et al., 2010	Mouse	Deregulated upon Reactive Oxygen Species neuronal stress	Lukiw and Pogue, 2007	Human	Plasma, urine, CSF	Melkonyan et al., 2008; Alexandrov et al., 2012; Sheinerman et al., 2012; Patz et al., 2013	Human
miR-134	Synaptic development, maturation and/or plasticity	Gao et al., 2010	Mouse	Detected in MCI human blood plasma	Sheinerman et al., 2012	Human	Plasma, urine, CSF	Melkonyan et al., 2008; Sheinerman et al., 2012; Pacifici et al., 2013; Patz et al., 2013	Human
miR-137	Neuronal maturation and dendritic morphogenesis during development	Taylor and Gercel-Taylor, 2008; Szulwach et al., 2010; Fang et al., 2012	Human, mouse, rat (PC12)	Affects Aβ generation	Szulwach et al., 2010; Geekiyanage and Chan, 2011	Mouse, human	Plasma, urine, CSF	Melkonyan et al., 2008; Geekiyanage et al., 2012; Pacifici et al., 2013	Human

Extracellular miRNA originating from specific tissues such as the brain and cancerous tissues can be released into biological fluids for example, Cerebral spinal fluid (CSF) (Cogswell et al., 2008; Baraniskin et al., 2012b), blood (Hunter et al., 2008; Baraniskin et al., 2011), saliva (Patel et al., 2011; Ogawa et al., 2013) and urine (Gilad et al., 2008; Qi et al., 2012). Despite the presence of high RNase activity, circulating miRNAs are protected from degradation by binding to RNA binding proteins such as lipoproteins (Arroyo et al., 2011; Vickers and Remaley, 2012) or contained in membrane derived microvesicles, in particular, exosomes (Mitchell et al., 2008; Arroyo et al., 2011). Exosomes are formed within multi-vesicular bodies (MVBs) in the endosomal system, which co-ordinates the transport of cargo between the plasma membrane, trans-Golgi network (TGN) and lysosomal system [reviewed in (Bellingham et al., 2012b)]. Exosomes can function as an intercellular delivery mechanism by sending miRNA cargo to initiate cell-to-cell communication as they shuttle between neighboring and distant cells (Valadi et al., 2007).

Profiles of deregulated miRNA isolated from peripheral blood (Jin et al., 2008) and serum (Skog et al., 2008; Taylor and Gercel-Taylor, 2008; Edbauer et al., 2010) have been demonstrated and suggest they have diagnostic potential for human diseases such as cancer. For example, the pathology of various cancers and miRNA originating from the site of metastasis correlate positively in both plasma and serum (Skog et al., 2008; Tsujiura et al., 2010). The use of miRNA expression levels as biomarkers can be applied to other human diseases and has not been thoroughly investigated in neurodegenerative diseases such as AD. There are some limitations to this approach, in particular the transport of brain specific miRNA through the blood brain barrier (BBB) into the circulation system of which the current mechanism is unknown. The BBB serves as a tight control point that has specialized molecular machinery to regulate the transport of nutrients and macromolecules, while ensuring viruses and bacteria do not cross the barrier (Begley and Brightman, 2003). On the contrary, under normal conditions, Aβ is cleared from the brain and transported across the BBB mediated by low-density lipoprotein receptor-related protein (Tanzi et al., 2004), suggesting the possibility of other neurodegenerative disease markers crossing the BBB.

There are two possible mechanisms by which miRNAs are able to transport through the BBB. Firstly, a number of neurological diseases such as Multiple Sclerosis and meningitis are well known conditions that weaken and eventually disrupt the BBB, consequently permitting a non-specific release of cellular factors and nucleic acids (Begley and Brightman, 2003). Moreover, thinning and perforations of the vascular basement membrane have been observed in post-mortem brains of late-stage AD patients (Blennow et al., 1990; Berzin et al., 2000; Zipser et al., 2007). Secondly, exosomes and microvesicles may play an important role as carriers of miRNA across the endothelial cellular layers of the BBB in order to communicate between the brain and distant organs via biological fluids (Haqqani et al., 2013). A proposed mechanism involves the transcytosis of extracellular vesicles, such as exosomes, across endothelial cells of the BBB by receptor-mediated endocytosis and releasing exosomal contents into circulation. As a result, the contents of the vesicles can be used as biomarkers reflective of the brain (Haqqani et al., 2013). miRNAs found highly abundant in the brain have been detected in human biological fluids such as plasma, urine, and CSF (summarized in Table 1).

### Detection of microRNA in blood

Blood is a highly reliable specimen used for diagnostic testing, with the majority of blood tests being minimally invasive. The cellular components of blood (red blood cells, white blood cells and platelets) provide a rich source of RNA species suitable for biomarker analysis. The most abundant source of miRNAs in blood are found in white blood cells (WBCs). The analysis of miRNA in cellular components of the blood may provide an understanding into the indirect causes of neurodegeneration or indeed reveal information on the pathogenesis of sporadic AD (Schipper et al., 2007; Xu et al., 2007). However, as the brain derived miRNA signal is essentially diluted in the circulating blood, there lies a greater signal-to-noise ratio.

In order to detect disease-specific miRNA profiles, the analysis of plasma and serum (cell-free) samples is most commonly performed (Chen et al., 2008; Pareek et al., 2011; Turchinovich et al., 2011), whereby a smaller but specific pool of miRNAs can be detected. The appeal in detecting circulating miRNA profiles is the potential of capturing the intracellular cross-talk between neighboring and distant cells in the body (Valadi et al., 2007; Turchinovich et al., 2011; Jones et al., 2012). Only a handful of studies have profiled miRNA biomarkers in AD patients using plasma and serum samples. The expression levels of brain-enriched miRNAs (miR-137, miR-9, miR-29a, and miR-29b) have been found to be significantly down regulated in plasma collected from probable AD patients (Geekiyanage et al., 2012). In another study, miR-128 and miR-134 were found to be highly abundant in the brain, and were also detected in cases of mild cognitive impairment (MCI), which is an early form of AD (Sheinerman et al., 2012). Furthermore, it is possible to isolate exosomes from plasma and serum to profile exosomal-specific miRNA by differential ultracentrifugation (Skog et al., 2008; Taylor and Gercel-Taylor, 2008).

### Detection of microRNA in CSF

Identifying circulating miRNA biomarkers in blood represents a clinical advantage for early disease diagnosis however, differential miRNA expression may not accurately reflect miRNA deregulation in neuronal tissues subject to neurodegenerative disease. CSF is a clear biological fluid produced in the choroid plexus of the brain, and circulates though the inner ventricular system, across the BBB and is absorbed into the bloodstream. CSF represents a more suitable and relevant source of material for diagnosis of central nervous system (CNS) disorders. CSF is obtained by lumbar puncture and has been shown to contain circulating miRNAs that have been utilized in several studies for miRNA profiling of neurological and neurodegenerative disorders; including AD (Cogswell et al., 2008; Alexandrov et al., 2012; Lehmann et al., 2012), schizophrenia (Gallego et al., 2012), Multiple Sclerosis (Haghikia et al., 2012), HIV-encephalitis (Pacifici et al., 2013), traumatic brain injury (Balakathiresan et al., 2012; Patz et al., 2013) and various cancers of the CNS (Baraniskin et al., 2012a,b; Teplyuk et al., 2012).

Studies of CSF from AD patients have used either a combination of miRNA microarrays (Alexandrov et al., 2012; Lukiw et al., 2012), multiplex miRNA qPCR assay (Cogswell et al., 2008) or a target candidate miRNA approach (Lehmann et al., 2012) to identify differentially expressed miRNA. In these studies, no correlation was observed between independent research groups or when validated in corresponding tissues samples extracted from AD patients (Cogswell et al., 2008). Using microarrays and qPCR validation, miR-9, miR-146a and miR-155 were found to be significantly up-regulated in AD patient CSF compared to age-matched controls (Alexandrov et al., 2012; Lukiw et al., 2012). However, these miRNAs were not identified in an independent study in which 60 miRNAs were found to be deregulated in CSF and corresponding brain tissue from AD patients (Cogswell et al., 2008). Likewise, candidate miRNA let-7b found increased in AD CSF (Lehmann et al., 2012) was not significantly altered in previous studies (Cogswell et al., 2008; Alexandrov et al., 2012; Lukiw et al., 2012). These observations highlight the need for a uniform approach to miRNA profiling for disease diagnosis. Collection of brain tissue, CSF, and peripheral blood samples in the same subjects would be advantageous however, this approach is challenging due the difficulty in recruiting study participants willing to undergo multiple invasive procedures.

### Detection of microRNA in urine

Clinically, urine is collected non-invasively for biomarker discovery and diagnostic purposes. The procedure for urine collection is relatively time- and cost-efficient compared with other clinical samples such as blood and CSF. This has led to an increase in miRNA biomarker studies examining urine samples to screen for disease biomarkers (Weber et al., 2010; Bryant et al., 2012; Wang et al., 2012a). Circulating extracellular miRNAs can be delivered to renal epithelial cells and released into the urine bound to RNA-binding proteins or packaged into microvesicles such as exosomes (Weber et al., 2010). The urine sediment, including whole cells, cell debris and polymerized protein, is able to be separated from whole urine using low-speed centrifugation (Wang et al., 2010, 2012a). Many studies exploit the urine cellular sediment obtained from low speed centrifugation to analyse miRNA implicated in prostate and bladder cancers (Wang et al., 2010, 2012c). Bryant et al. have reported several deregulated miRNAs associated with prostate cancer which were validated in serum, plasma and urine (Bryant et al., 2012). Analysis of urinary miRNA from the cell sediment may not be suitable for neurodegenerative diseases, as it is rich with cells or cell debris of hematologic origin, renal epithelial origin and urothelial origin in addition to microorganisms such as bacteria and yeast (Koss and Sherman, 1984; Wang et al., 2010). Fewer studies have analysed cell-free urine to isolate miRNAs, mainly because it may contain miRNAs from organs of the body outside of the excretory system. Exosomes can be purified from urine using a differential ultracentrifugation method which is the most widely applied technique (Alvarez et al., 2012). Future advances in methodologies to improve sensitivity and accuracy in profiling urinary miRNA biomarkers, in particular from using exosomes, would be of great value to investigate whether miRNA can be detected in urine.

Many studies using human cell lines differentiated with all-trans-retinoic acid, cultured primary neurons, astrocytes and brain sections from mice and human models highlight the significance of miRNA in neurodegeneration (Sempere et al., 2004; Smirnova et al., 2005; Alvarez et al., 2012). Overall, the potential to detect miRNA in biological fluids, in particular those highly expressed in the brain, is well supported by research published in the literature (summarized in Table 1). The majority of experimental methods have used Northern blotting, qPCR and microarrays though these methodologies are not suitable for biomarker discovery or mass screening programs. Researchers are now developing high-throughput, cost effective strategies to improve the sensitivity and specificity of for miRNA diagnostics in biological fluids. One of these technologies is deep sequencing.

## The use of deep sequencing technology to screen for microrna biomarkers

Implementing deep sequencing technology represents a powerful and innovative approach to discover differentially expressed miRNAs in neurodegenerative diseases (Brase et al., 2010; Debey-Pascher et al., 2012). The advantage of using deep sequencing, unlike traditional Sanger sequencing, lies upon the capability to simultaneously process millions of independent sequencing events. This offers billions of nucleotide information within a single experiment (Shendure et al., 2004; Church, 2006). Deep sequencing experiments enable comprehensive analyses of large amounts of sequence data, resulting in dramatically accelerated research compared to traditional labor-intensive efforts and is a powerful approach to determine accurate encoded-information from nucleotide fragments (Hall, 2007; Shendure and Ji, 2008; Tucker et al., 2009).

### Large-scale deep sequencing platforms

Detecting low abundance or differentially expressed circulating miRNA signatures in biological fluids requires a large-scale and high-throughput platform. The large-scale sequencing instruments presently available are the 454 Genome Sequencer (GS) FLX+ system from 454 Life Sciences, 5500 Sequencing by Oligo Ligation Detection (SOLiD) system from Applied Biosystems (Life Technologies), HiSeq 2000 system from Illumina and the recently introduced Ion Proton from Ion Torrent (Life Technologies). Each platform employs different sequencing chemistries for data generation (summarized in Table 2). All 454 systems adopt a principle of pyrosequencing which, is based on the detection of pyrophosphate molecules during nucleotide incorporation and the intensity of signals produced by chemiluminescence (Ahmadian et al., 2006; Rothberg and Leamon, 2008). SOLiD utilizes ligation-based chemistry with dye-labeled probes, involving rounds of oligonucleotide ligation extension and two-base encoding detection (Pandey et al., 2008). Illumina systems rely on a sequencing-by-synthesis (SBS) approach involving cycles of nucleotide incorporation and use of reversible dye terminators (Fuller et al., 2009). Ion Proton also employs SBS chemistry however, it measures signals through the production of hydrogen ions resulting from the process of nucleotide replication on a chip containing an array of semiconductor sensors (Rothberg et al., 2011). Despite the various sequencing chemistries, all large-scale deep sequencing platforms are capable of generating up to 3 billion sequencing reads, with an output of 0.7–600 gigabases (Gb), quickly and efficiently (Table 2). To date, many unbiased miRNA biomarkers have been discovered through large-scale deep sequencing techniques (Maes et al., 2009; Cortez et al., 2011; Etheridge et al., 2011; Bellingham et al., 2012a). However, deep sequencing of small sample sizes via large-scale platforms will be very expensive if the capacity of each run is not fully utilized (Wall et al., 2009). Furthermore, the advantages of these large-scale platforms are immediately offset by the reagent costs, in the thousands of dollars per sequencing run, and run times of up to 14 days, which are usually constrained in small laboratories and particularly in diagnostics (Morozova and Marra, 2008). In order to facilitate access of deep sequencing for the majority of laboratories, the recent launch of small-scale benchtop deep sequencers has offered a cheaper alternative to sequence genomes with greater speed, ~2 h run time, and lower cost compared to large-scale deep sequencer (Eyre et al., 2012).

**Table 2:** **Specifications of current “Next-Generation” Deep Sequencing platforms^†^**.

**Sequencing platform**	**Read length (bases)^#^**	**Throughput / time per run**	**Accuracy (%)**	**Advantages**	**Disadvantages**	**Cost per run**
Illumina	35–100	100–600 Gb / 2–11 days	98.0	Ultra high throughput	Short read assembly may miss large structural variations	$$$
HiSeq™ 2000				High capacity of multi-plexing		
					Signal interference among neighbouring clusters	
					
					Homopolymer errors	
Applied Biosystems	35–75	120 Gb / 7–14 days	99.9	Ultra high throughput	Short read assembly may miss large structural variations	$$$
				Two-base coding (higher accuracy)		
5500 SOLiD™					Long run time	
				High capacity of multi-plexing	Signal interference among neighbouring clusters
					Signal degradation	
Roche (454 Life Sciences)	700–1000	0.7 Gb / 0.35–0.42 days	99.9	Long read assembly allows detection of large structural variations	Lower throughput	$$
					Homopolymer errors	
					Signal interference among neighbouring clusters	
GS FLX Titanium				Short run time			
				
Ion Torrent	100–400	10 Gb / 4 h	98.5	Fast run time	Newest to the market	$/$$
Ion Proton™	100–400	30 Gb / 4 h		Highly scalable (different chips available)		
PI						
PII				Low cost		
Ion Torrent	100–400	0.01 Gb / 1 h	98.5	Highly scalable (different chips available)	Homopolymer errors	$
Ion PGM™	100–400	0.1 Gb / 2 h			
314 chip	100–400	1 Gb / 3 h		Low cost		
316 chip				Fast run time		
318 chip						
Illumina	35–150	1.5 Gb / 27 h	99.2	Well-proven sequencing technology	Low abundance of amplified template	$
MiSeq™						
				Fully automated workflow		
				Low cost		
				Fast run time		
Roche (454 Life Sciences)	250–400	0.035 Gb / 8 h	99.0	Long read length	Lower throughput	$
				Relatively fast run time	Homopolymer errors	
454 GS Junior						

# * Average read length depends on specific sample and genomic characteristics*.

† * Specifications for all platforms are derived from company websites*.

### Benchtop deep sequencing platforms

The smaller benchtop deep sequencing instruments allow the possibility of small-scale biomarker discovery. Commonly used small-scale sequencing instruments are 454 GS Junior from 454 Life Sciences, MiSeq from Illumina, Ion Personal Genome Machine (PGM) from Ion Torrent (summarized in Table 2). The employment of benchtop sequencers offers an alternative option for low number of samples in a cohort (Kumar and Webster, 2011) and present additional advantages with their convenience of storage and simple procedures in sample preparation (Hutchison, 2007; Eyre et al., 2012; Wilson, 2012). Loman et al. and Quail et al. sequenced bacterial genomes, using laser-printer sized benchtop platforms capable of generating usable sequences with fast turnaround time in a straightforward workflow and low running costs. In early 2010, the release of 454 GS Junior instrument substituted the larger 454 GS FLX instrument for small-scale sequencing. It employs similar template amplification and pyrosequencing-based approaches as of the 454 higher-scale sequencer (Margulies et al., 2005). Illumina and Ion Torrent also provide for large and small-scale projects with the introduction of Illumina MiSeq and Ion PGM, smaller versions of the Illumina HiSeq and Ion Proton, respectively. These exploit the existing platform-specific sequencing chemistry suitable for smaller scale projects (Bentley et al., 2008). All benchtop sequencers are as competitive as their respective large-scale platforms as they are able to generate millions of reads in their output (Loman et al., 2012a; Quail et al., 2012). Although both 454 JS Junior and Illumina MiSeq utilize years-proven sequencing strategies, the Ion PGM is a useful addition to the current deep sequencing platforms by adding scalability (i.e., different chips to allow different scale of studies to be sequenced cost-efficiently) and lower instrument cost (Defrancesco and Subbaraman, 2011; Scholz et al., 2011; Kodama et al., 2012; Vogel et al., 2012). The output of sequencing reactions ranges from 10-, 100-, to 1000-million reads due to the availability of three different chips used for sequencing (e.g., 314-, 316-, and 318-chips). The signal detection of each nucleotide in Ion Torrent system depends on a sensitive pH measurement, thus eliminating the requirement for modified chemiluminesecent reporters and expensive detector devices (Pareek et al., 2011). In essence, benchtop deep sequencing technologies provide an affordable way to produce accurate throughput with sufficient sequencing coverage. Notably, Ion Proton can be classified as benchtop sequencer as it is currently the only instrument promised to provide shorter sequencing time than other large-scale systems and hundred folds more data output than other bench-top sequencers. Once these advantages are tested in future studies, Ion Proton could have its own niche bridging between small-scale and large-scale for routine full genome screening.

## Biomarker discovery to clinical practice

Typically, the data generated from benchtop deep sequencing instruments (e.g., high assembly coverage by 454 GS Junior due to long read length) are generally sufficient to obtain disease specific profiles from individual samples (Loman et al., 2012b). Ultimately, the choice of platform will depend on its performance metrics (i.e., read length, accuracy and data output) to complement the type of study being undertaken. For clinical practice, platforms such as the Ion PGM and MiSeq offer the best value for money, more flexibility, accuracy, adequate throughput and coverage depths for study of miRNA.

**Figure 1:**
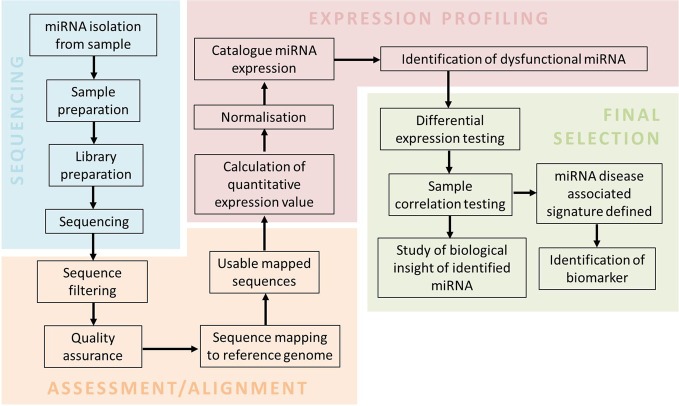
**A miRNA-sequencing workflow**. The workflow consists of sample preparation, sequencing, assessment and alignment, expression profiling and final selection. Sequencing involves sample preparation of isolated miRNAs to generate a library for the sequencing reaction. Assessment and alignment requires bioinformatics tools to generate usable mapped sequences. Final selection of miRNAs related to the disease are filtered by sophisticated statistical tools.

To incorporate miRNA deep sequencing for clinical practice, a simple and standardized workflow for the routine biomarker and diagnostic screening needs to be defined (Barzon et al., 2011; Natrajan and Reis, 2011; Radford et al., 2012; Rizzo and Buck, 2012). A typical workflow involved in performing miRNA-sequencing from sample preparation to data analysis is outlined in Figure 1. The sequencing workflow consists of the following steps: (i) miRNA isolation; (ii) Library preparation and enrichment of templates containing size selected libraries of the appropriate fragment length; (iii) and Sequencing reaction. Isolation of low abundance miRNA present in body fluids (e.g., blood and urine) can be obtained by the use of RNA isolation columns that specifically enrich for miRNA (Mitchell et al., 2008; Arroyo et al., 2011). Preparation of isolated miRNA for sequencing includes the assessment of quality (i.e., RNA integrity number; RIN), profiling and quantification of small RNA species, in particular miRNA (Ozsolak and Milos, 2010). Preparation of sample library often requires barcoding, which involves ligation of different adaptors of identifiable sequences to either end of each sample. This method is to increase capacity as it allows multiplexing of samples in the same sequencing reaction (Chen et al., 2012). Template preparation for sequencing and the sequencing reaction is platform specific as seen in Table 2.

Another approach to increase throughput is by capturing and sequencing disease specific targets known as sequence enrichment. In clinical practice, the factors to consider while sequencing for an enriched population of targets may involve the size of the region of interest, capture efficiency of interest region, average read depth coverage of the targeted region, distribution of coverage and its sensitivity and specificity (Mamanova et al., 2010). The quality control involved would require a set of positive and negative control references to establish a standard depth and quality percentage of targeted regions for determining the accuracy of enrichment (Harismendy et al., 2009; Teer et al., 2010). Furthermore, reference controls eliminate any experimental problems which may arise from sequence variations that affect hybridization to biotinylated probes during enrichment, causing low capture efficiency (Schwartz et al., 2011). Hence, quality control measures should be routinely assessed before proceeding to analyse samples for a diagnosis to eliminate false positives which may be caused by poor miRNA quality or technical artefacts (Fehniger et al., 2010; Lee et al., 2010). Furthermore, instruments that provide a fast turn-around time will be suitable in diagnostic laboratories in order to produce diagnostic reports in a timely manner (Tucker et al., 2009). In clinical practice, a high throughput strategy may involve using deep sequencing platforms to screen for AD biomarkers by indexing a large number of patient samples and pooling samples into one sequencing run. Indexing patient samples and batch testing in a diagnostic laboratory would be a cost-effective and practical approach to handle large population screening.

All deep sequencing instruments produce a massive quantity of raw data that requires extensive computational tools to process the information (Zhang et al., 2011). Open-source tools and in-house (Perl) scripts are available for handling large quantities of sequencing data by providing an integrated and streamlined analysis workflow (Nix et al., 2010). A fundamental workflow for post-analytic miRNA data analysis (Figure 1) consists of four fundamental steps: (i) Sequence assessment; (ii) Sequence alignment; (iii) Profiling of miRNA associated with neurodegenerative disease; (iv) and Final selection. This workflow requires databases and tools such as FastQC (quality control check), miRbase/miRandola (miRNA database), bowtie (short read aligner) and EdgeR/DESeq (normalization) (Horner et al., 2010; Kozomara and Griffiths-Jones, 2011; Russo et al., 2012). In order to make data analysis more accessible to the diagnostic end-user, there are a number of commercially available software packages that aim to make analysis uncomplicated by providing user-friendly graphical interfaces (Richter and Sexton, 2009). This includes Partek Genomics Suite, CLC Genomics Workbench, Ingenuity Systems and platform-specific software (e.g., Ion Torrent Suite and Illumina GenomeStudio). These commercial software tools have less flexibility and scalability in terms of their parameters' settings, but are suitable for small-scale studies to establish miRNA profiles and perform differential expression analysis in order to detect disease specific miRNA (Meldrum et al., 2011).

## Conclusions

Early diagnosis of AD is critical as it is hypothesized that the pathology of the disease occurs up to 20 years before cognitive decline. Many groups have searched for protein biomarkers in blood, plasma, serum and CSF have not yielded a reliable, sensitive, and specific candidate biomarker marker for AD diagnosis. Due to the sensitivity of deep sequencing, it is possible to detect genetic modifications in disease, in particular deregulated miRNA. The possibilities of profiling miRNA in CSF, urine, blood, plasma and serum have been explored with some successes in cancer and may be applicable to AD and other neurodegenerative disorders. In addition, the genetic information contained in circulating exosomes may provide a highly specific readout of disease and should be further investigated. Deep sequencing of miRNA together with high-throughput validation methods will complement diagnostic testing and represents a vital step toward developing a cost-effective, non-invasive and low risk diagnostic test to detect the onset and monitor various stages of AD. Furthermore, the development of a diagnostic test comprising of a profile of RNA biomarkers associated with AD, and has potential for other neurodegenerative diseases such as Parkinson's, Prion and Huntington's diseases.

### Conflict of interest statement

The authors declare that the research was conducted in the absence of any commercial or financial relationships that could be construed as a potential conflict of interest.
